# Magnitude and possible risk factors of musculoskeletal disorders among street cleaners and solid waste workers: a cross-sectional study

**DOI:** 10.1186/s12891-023-06619-z

**Published:** 2023-06-15

**Authors:** Melkamu Alie, Yohannes Abich, Solomon Fasika Demissie, Fkrte Kebede Weldetsadik, Tesfa Kassa, Kassaw Belay Shiferaw, Balamurugan Janakiraman, Yohannes Awoke Assefa

**Affiliations:** 1 Department of Physiotherapy, School of Medicine, College of Medicine and Health Sciences, University of Gondar, P.O. Box: 196, Gondar, Ethiopia; 2 SRM College of Physiotherapy, SRM Institute of Science and Technology (SRMIST), Kattankulathur, Chennai, Tamil Nadu India; 3 Department of Occupational Therapy, School of Medicine, College of Medicine and Health Sciences, University of Gondar, Gondar, Ethiopia

**Keywords:** Musculoskeletal disorders, Street sweepers, Solid-waste, Risk factors

## Abstract

**Background:**

In the absence of a standardized work environment, insurance system, occupational safety measures and expanding workload an uptrend of musculoskeletal disorders secondary to occupational hazards is observed among a wide range of occupations in developing countries including street sweepers/cleaners. The aim of this study is to determine the burden and potential factors associated with musculoskeletal disorders among street cleaners/solid waste collectors in Gondar town, Ethiopia.

**Methods:**

A cross-sectional study design was used to determine the burden and identify potential risk factors of musculoskeletal disorders among street cleaners. Street cleaners (*n* = 422) working experience of at least one year were randomly selected from the community at their respective work sites (street). A face-to-face interview recorded the participant’s response addressing socio-demographic, occupational, job satisfaction, disability related to basic ADL, physical measurements, and self-reported pain using the Nordic-Musculoskeletal questionnaire. The logistic regression model was created to identify potential factors associated with self-reported MSDs.

**Results:**

The sample consists of women street sweepers/cleaners (100%, *n* = 422, response rate 100%) with at least one-year of work experience with a mean age of 37.03 ± 8.26. About 40% of women sweepers were illiterate and 95% reported no job satisfaction. The overall prevalence of MSDs was 73% (*n* = 308, 95% CI; 68.5, 77.2), among them nearly 65% reported having experienced disability in performing basic ADL in the past 12 months. Low back pain was the most prevalent region (*n* = 216, 70.1% case versus MSDs *n* = 308). In univariate and multivariate logistics analysis, being overweight/obese (AOR of 4.91 (95%, 2.22, 10.87)), age group 35 and above (AOR 2.534 (1.51, 4.26)), not-satisfied with job (AOR 2.66 (1.05, 6.75)), and street cleaning distance of longer than 2 km (AOR 2.82 (1.64, 4.83)) were significantly associated with self-reported musculoskeletal disorder..

**Conclusion:**

This study demonstrated higher self-reported MSDs among street sweepers/cleaners. Modifiable predictors like overweight, lack of job satisfaction, and cleaning longer distance were identified to be associated. Hence, there is a need for ergonomic measures and policy to curb these factors to reduce the burden of MSD among women street sweepers.

## Background

Street cleaning services are an integral part of the waste management system and a clean environment [1]. An enormous increase in population, urban land, households, utilities, lack of stakeholder cooperation, and weak institutional capacity has resulted in over-pressure on the waste management system and street cleaners in the Sub-Saharan region, particularly Ethiopia [1, 2]. The occupation of street cleaning is a vigorous job demanding continuous repetitive physical tasks, further in the absence of an automated facility, safety measures, and a work-rest schedule street cleaning job can be hazardous.

Numerous literature has reported the occupational exposure of street cleaners to pollutants or hazardous materials and the development of diseases like; asthma, chronic respiratory diseases, eye irritations, skin conditions, stress, and hypertension [3–7]. Musculoskeletal disorders (MSDs) are the most common non-fatal and very disabling health problem among street cleaners. Work-related musculoskeletal disorders are one of the foremost reasons for health concerns and global disability with a significant impact on productivity, the economy, and individual health [8]. Though there is an increasing trend of the global burden of MSD in recent years, MSDs are rarely fatal and assumed irreversible and have received partial attention by researchers, policymakers and lesser prioritization of health resources in many developing countries. An estimated 1.71 billion people were affected by musculoskeletal disorders globally, DALYs were 150.1 million based on GBD 2019 and the utmost bodily region (67%) reported was LBP. MSDs are ranked first in years lost due to disability (YLDs) and it is one of the most common reasons for seeking rehabilitation services among adolescents and adults [8–11]. Though the prevalence of MSDs is higher in developed countries, the DALYs have shown a downtrend due to improved informed policy and implementations.

The global burden and trends of MSD have varied greatly between different countries, and Socio-demographic Index (SDI). Reporting the burden of disease, among varied occupations, regions, SDI and constant examining of risk factors can immensely improve the understanding of the burden and potential factors associated which will surely help to better characterize, and prevent MSDs related to work [8]. A study of spatial–temporal distribution [11] of MSD (based on GBD 2019) across 204 countries and 21 sub-region reported that the overall average prevalence per sub-regions ranged between 0.09% (for OA hand) and 15.8% (LBP), in Southeast Asia and in high-income North America respectively. Sub-Saharan Africa (SSA), sub-region observed the lowest overall prevalence for all years combined at 3.9% (LBP), 1.4% (knee OA), and 0.7 (neck pain) in Eastern and Central SSA. Notably, GBD 2019 reported that occupational risk demonstrated the highest contribution to MSD followed by tobacco use and high BMI [9], and also highlighted that the scarcity of evidence on the burden of MSD beyond western and Asian countries would have led to under-estimation in SSA with the larger increase in SDI. Unlike the developed countries, it is rather challenging to estimate the burden of work-related MSDs in developing countries with the absence of health insurance, medical claims, occupational-health center documentation, and lack of large-scale population studies [8, 12, 13].

In the study (Amhara, Ethiopia) region, street cleaning, and garbage collection are done on a manual basis and then manually loaded in trunks, women from low education and socio-economic strata are exclusively or more likely to participate in this job [2]. Studies globally reported higher prevalence, ranging from 49% to 85 [12–17], and suggestive of an increased probability of developing MSDs among street cleaners. Regional studies reporting morbid MSD among street cleaners are scarce and hardly any regional studies have assessed the occupational exposure to the development of MSD and the potential risk of MSD. The present study is aimed to determine the burden of musculoskeletal disorder, assess the work exposure to the development of MSD, and identify factors associated with street cleaners.

## Methods

### Study design, period, and setting

The study used a cross-sectional design to determine the burden of MSD and factors potentially associated with street cleaners. The study was conducted from February to June 2022 and reported in accordance with the STROBE (STrengthening the Reporting of OBservational studies in Epidemiology) [18] recommendations. The proposal of the study was presented to the Ethical Review. Board of School of Medicine, University of Gondar, and ethical approval was secured. Further, permission to collect data from street sweepers/cleaners was obtained from the central Gondar town administrative zone, solid-waste management office. A written informed consent was obtained from all the prospective participants, thumb impression was taken from those (illiterates) who cannot do so after reading the consent form, and this study was conducted in accordance with the declaration of Helsinki.

The Gondar town is situated 728 km from the capital city (Addis Ababa) of the country, at an altitude of 2706 m above sea level. According to 2007 census by Central Statistical Agency (CSA) of the Ethiopia, the North Gondar zone had an estimated population of 2,929,628 inhabitants with 654,803 households. Gondar is a rapidly growing and urbanizing town. The rapid expansion in population has resulted in increased volumes of solid trash and increased infrastructural demand. Further, the mountain terrain topography of Gondar comprising scattered hills, and valleys possess unique challenges for street cleaners/sweeper [2].

### Source and study population

The source population was all the street cleaners/sweepers and solid waste collectors employed in the administrative bureau of Gondar town and engaged in street-based solid-waste collection activities. The study population was all the street cleaners/sweepers and garbage collectors employed in the administrative bureau of Gondar town and engaged in street-based waste collection activities in Gondar town working at least for one year. About 902 women were street cleaners/sweepers were currently employed in the Gondar town and 828 had a minimum of 1-year work experience. Street sweepers aged 18 years and above, both genders, with a minimum of 1-year work experience which would allow representation of participants who had worked in different sites, and seasons, and working in the study site were included. Using a screening checklist the sweepers/cleaners with known neuromuscular disorders, pregnant, and those in the supervisor category were excluded. Employees who are involved in jobs like driving motorized or manual solid-waste vehicles were also excluded. During the study proposal we intended to include sweepers of both genders, but none of the male participant working in solid waste management fitted into our operational definition for street sweepers/cleaners.

### Sample size determination

The power calculated sample required for this study was estimated using the formula [19] for single population proportion and the following assumptions; a 5% level of significance, an expected frequency of MSDs of 50% was used since there were lack of similar regional studies, *Z* = 1.96 critical values with 95% CI (confidence intervals), and a marginal error of 5% (d). The equated power sample was 384 and assuming a 10% contingency margin including the non-responses, the final sample size was 422.

### Sampling technique and procedure

The administrative office was contacted and a list of 828 workers (≥ 1-year experience) allotted to 13 (sites) specific administrative areas/zone (kebele) was obtained. Since, the proportions of allocation of workers were different based on the number of households in the kebele. We used a proportional sampling for all 13 sites to achieve the final sample of 422. In total, 783 eligible voluntary respondent were invited and of them all the female workers agreed to participate. At the site level, a simple random using lottery method was used to recruit the participants (*n* = 422).

### Variable definition and data collection procedures

Street cleaners/sweepers or waste collectors are defined as those workers in contract with administrative zone, solid-waste management office and involved in the cleaning of streets, collecting garbage from households, institutions, and roads, using brooms, dustpans, or plastic garbage bags.

Participants who self-reported experiencing pain in one more of the nine anatomical regions (neck, upper back, shoulders, elbows, wrists/hands, lower back, hips/thighs, knees, ankle, and feet) [20] in the past 7 days and 12 months before the survey were considered to have a musculoskeletal disorder. The respondents, who reported “difficulty” or “received assistance” or were “unable” to perform one or more of the basic activities of daily living (personnel hygiene /grooming, dressing, ambulating/transferring, eating, washing clothes, and toileting) [21] due to MSD in the past 12 months, were classified as disabled. The Minnesota Satisfaction Questionnaire (MSQ) [22] was used to measure the level of job satisfaction on a 1 to 5 Likert scale. The MSQ scale is a well-known and stable over time instrument with the coefficient alpha ranging from 0.85 to 0.92. The total score of 25 items MSQ, ranges from 25 – 100, and the job satisfaction of the respondent is categorized as; not satisfied (25 – 37.50), somewhat satisfied (37.6 – 62.50), satisfied (62.6 – 87.5), and very satisfied (87.6—100). In this study, the Cronbach’s α of the MSQ (*n* = 422) was 0.913.

Data was collected by a face-to-face scheduled interview method using a literature review-based structured questionnaire which also included a modified Standardized Nordic Musculoskeletal Questionnaire (NMQ) [20] to assess the MSDs. A body diagram with the arrow labeled bodily regions was used. Trained data collectors (3 physiotherapists) collected information regarding socio-demographic, clinical, occupational characteristics, and anthropometric measurements. On average, the interview time lasted ranged from 20 to 25 min. The questionnaire was pre-tested using 21 street cleaners (5% of the sample size) with similar characteristics to the study sample from another kebele. The Cronbach’s alpha coefficient in the pre-test was (*n* = 21) 0.87. The outcome tools (long form of MSQ and NMQ) used in this study were not available in the local language versions.

#### Data management and analysis

The questionnaire was checked for clarity and completeness. Data were coded and entered into Epi-info version 7.0 and then exported to the Statistical Package for Social Science (version 21) for Windows. A Chi-square test was conducted to describe the association of different categories with the MSDs (categorized as “yes versus no”). Descriptive statistics like percentage, frequency, mean, median, range, and standard deviation was used to describe the study population in relation to relevant variables, and findings were presented with texts, tables, and figures. To examine the association between the outcome variable (MSDs; “yes vs no”) and the predictor variables, the bi-variable logistic model (*p* < 0.25) was used. Predictor variables included in the regression model were; age (categorized 18—34 and ≥ 35 years), educational level (no formal schooling, primary and secondary), BMI (normal and overweight/obese), job satisfaction (no satisfied, somewhat satisfied, satisfied and very satisfied), work experience (< 10 and ≥ 10 years), cleaning distance/day (< 2 km and > 2 km), broomstick length (short versus long), and brook stick weight (< 800 gm and ≥ 800 gm). Independent variables which have significant associations with the dependent variable in bivariate logistic regression (*p* < 0.25) were entered into multivariate analysis to identify the important determinant factors of MSDs in road cleaners and help to control for possible confounding effects. Interaction terms were used (overweight/obese*age ≥ 35) to examine the potential association between predictor variables and MSDs. The Hosmer–Lemeshow goodness of fit test [23] for logistic regression was checked and the multi-collinearity was checked by the variance inflation factor (VIF) that was < 10. Finally, AOR with a 95% of confidence interval at the p-value of < 0.05 was reported as statistically significant.

## Results

### Characteristics of the participants

#### Socio-demographic

A total of 422 respondents consented to participate and completed the interview. The response rate of the participants was 100%. All of the participants were females 422 (100%) and the male employees were exclusively involved in the transport category, none of the male workers were involved in sweeping or cleaning the street, and hence not considered. The mean age of the participants was 37.28 years ± 8.77, and 275 (65.2%) participants were aged 35 years and above. About 2 out of 5 participants (40%) never had formal schooling. The majority of participants (54.7%) reported being single (10.7%), divorced (27.7%), and widowed (16.3%). Hundred and sixty-two (38.4%) respondents reported alcohol drinking habits, 97 of the 422 were overweight/obese, and none of them reported smoking habits. The distribution of socio-demographic characteristics is shown in Table 1. Overall, the participants were working for 5—6 days per week, and the mean monthly income was 19.5 US dollars.

**Table 1:** Socio-demographic, behavioral, physical measurements, job-related characteristics and distribution of musculoskeletal disorders (12 months, yes vs no) of street cleaners/sweepers (*n* = 422)

**Variables**	**Sample total**	**MSDs n (%)**		**Χ**^**2**^**/t**	***P***
	**n (%)**	**Yes**	**No**		
All participants	422 (100)	308 (73)	114 (27)		
^a^Age (years)	37.03 ± 8.26	38.22 ± 8.2	33.8 ± 7.55	4.54^t^	0.000
18 – 34	147 (34.8)	86 (27.9)	61 (53.5)		
≥ 35	275 (65.2)	222 (72.1)	53 (46.5)	23.99	0.000
Education level					
No formal schooling	172 (40.8)	133 (43.2)	39 (34.2)		
Primary	177 (41.9)	124 (40.3)`	53 (46.5)		
Secondary	73 (17.3)	51 (16.6)	22 (19.3)	2.77	0.25
Marital status					
Married	191 (45.3)	128 (41.6)	63 (55.3)		
Single	45 (10.7)	27 (8.8)	18 (15.8)		
Divorced	117 (27.7)	92 (29.9)	25 (21.9)		
Widowed	69 (16.4)	61 (19.8)	08 (7.0)	17.51	0.001
^a^Height in m	1.54 ± 0.06	1.54 ± 0.06	1.55 ± 0.06	2.19^t^	2.38
^a^Weight in kg	54.45 ± 6.9	55.1 ± 7.2	52.78 ± 5.85	3.11^t^	0.09
^a^BMI kg/m^2^	22.96 ± 2.66	23.32 ± 2.6	21.98 ± 2.33	4.7^t^	0.12
BMI					
Normal	325 (77)	219 (71.1)	106 (93)		
Overweight/obese	97 (23)	89 (28.9)	08 (07)	22.49	0.000
Alcohol habits					
Yes	162 (38.4)	127 (41.2)	35 (30.7)		
No	260 (61.6)	181 (58.8)	79 (69.3)	3.9	0.030
^a^Job satisfaction (MSQ score)	59.19 ± 2.55	58.9 ± 2.5	59.8 ± 2.4	3.25^t^	0.78
Job satisfaction					
Satisfied	23 (5.5)	10 (3.2)	13 (11.4)		
Not satisfied	399 (94.5)	298 (96.8)	101 (88.6)	10.74	0.003
Cleaning distance/day					
1–2 km	92 (21.8)	50 (16.2)	42 (36.8)		
3 km	167 (39.6)	122 (39.6)	45 (39.5)		
4 km	163 (38.6)	136 (44.2)	27 (23.7)	25.23	0.000
Change of brooms/month					
1-2	366 (86.7)	293 (95.1)	73 (64)		
> 2	56 (13.3)	15 (4.9)	41 (36)	69.8	0.000
Broom length					
115	335 (79.4)	247 (80.2)	88 (77.2)		
120	73 (17.3)	52 (16.9)	21 (18.4)		
125	14 (3.3)	09 (2.9)	05 (4.4)	0.74	0.68
Weight of the broom					
< 800 gm	292 (69.2)	212 (68.8)	80 (70.2)		
≥ 800 gm	130 (30.8)	96 (31.2)	34 (29.8)	4.11	0.44

Values are presented as number (%) or mean ± standard deviation indicated by ^a^Chi-square test was used for categorical variables, student-t-test for continuous variables

*BMI* Body Mass Index, *k*g Kilogram, *m* meters*, MSQ* Minnesota Satisfaction Questionnaire

#### Job and work-related instruments

All the participants were working for 5—6 days per week. Most of them (n 352, 83.4%) had less than 10 years of work experience. Only 19.2% (n 81) had taken occupational health safety training and the PPE utilization of face masks, gloves, aprons, boots, and reflector gowns were 14.5%, 32%, 37.2%, 10.7%, and 15.6% respectively. Among the overall participants, only 5.5% (n 23) were found to have job satisfaction. About 78% of the sweeper reported that the cleaning distance per day was 3 or 4 km and one among eight (13.3%, *n* = 56) changed their brooms more than twice in a month. The most common broomstick length used was 115 cm (79.4%, *n* = 335), followed by 120 cm and 125 cm by 17.3% and 3.3% sweepers respectively. About 70% of them used lighter broomsticks (< 800 g).

#### Musculoskeletal disorders among street sweepers, disability of ADL, and distribution

**Fig. 1:**
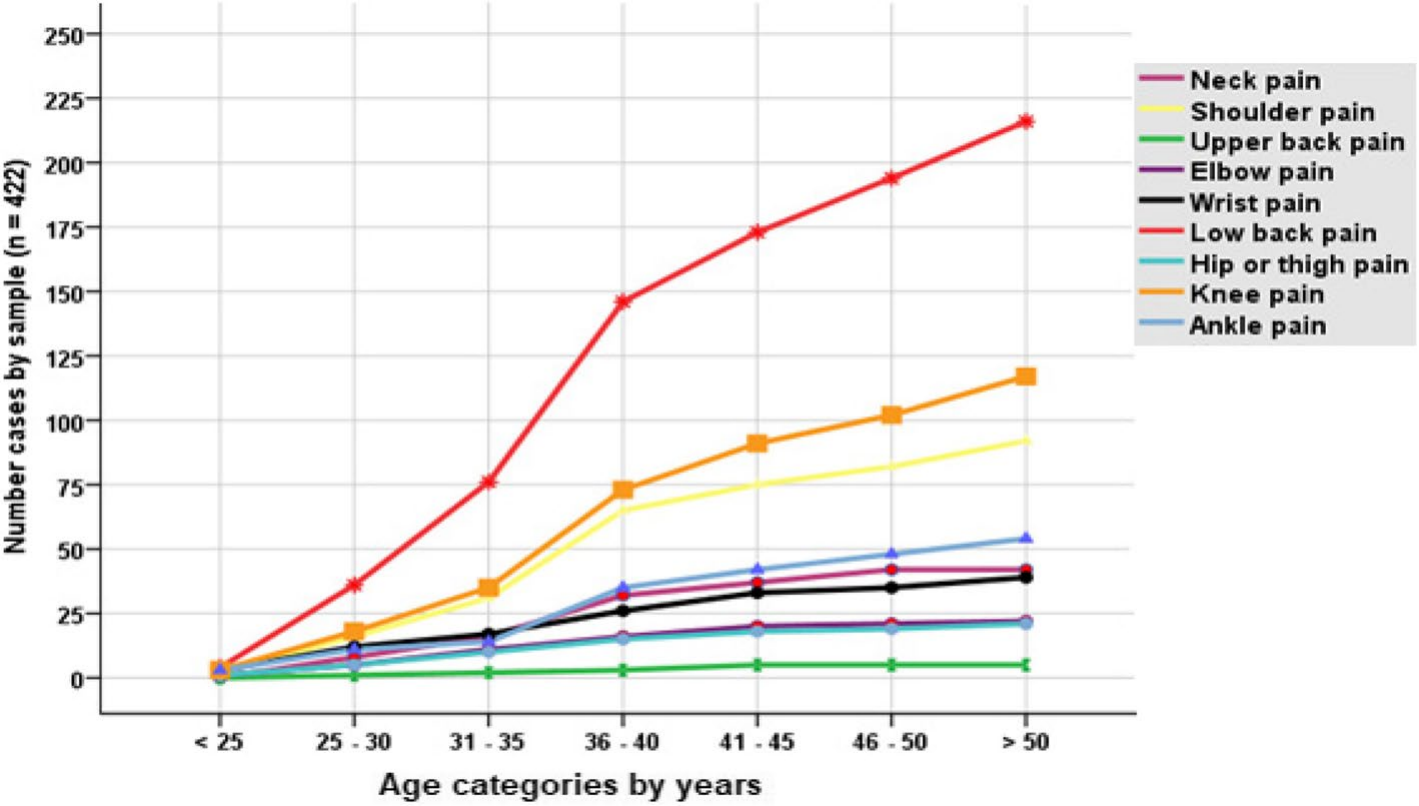
Frequency distribution of bodily regional pain by age-category

The overall prevalence rate of MSDs in street sweepers was 73% (*n* = 308, 95% CI; 68.5, 77.2) in the past 12 months and 59.7% (*n* = 252) in the past 7 days. A statistically significant difference was observed in the prevalence of MSDs between the age group (18 to 34 years 27.9% vs ≥ 35 years 72.1%; Χ^2^ (1, *n* = 422) = 23.9, *p* < 0.001, phi = 0.24). The distribution of MSDs was significantly higher among the normal-weight (BMI) sweepers (normal weight 71.1 vs overweight/obese 28.9%; Χ^2^ (1, *n* = 422) = 22.49, *p* < 0.001, phi = 0.25). The majority of married women sweepers reported MSDs 41.6%, followed by divorced 29.9%, widowed 19.8%, and single 8.8%. Longer cleaning distance covered in a typical day was significantly associated with the higher reporting of MSDs among the respondents (4 km 44.2%, 3 km 39.6%, vs 16.2%; Χ^2^ (2, *n* = 422) = 25.23, *p* < 0.001, phi = 0.33). The number of brooms changed in a month was significantly associated with the experience of MSDs (> 2 brooms/month 4.9% versus 1 to 2 brooms in a month 95.1%, Χ^2^ 69.8, *p* < 0.001). The frequency of MSDs case increased with age and higher frequency was observed in the age category of 46 – 50 years and above 50 years (Fig. 1). The most common regional pain reported were low back (n 216), knee (n 117), and shoulder (n 92). All the participants (n 422) responded ‘tiresome and challenging’ for the attribute question “how would you describe your work”.

The bodily region pain among the respondents who reported MSDs (n 308) was low back pain (70.1%), knee pain (38%), shoulder pain (29.9%), ankle/foot pain (17.5%), neck pain (13.6%), wrist/hand pain (12.7%), elbow pain (7.1%), hip/thigh pain (6.8%), and upper back pain (1.6%). About 65% of those who self-reported MSDs were observed to have suffered a disability in performing basic ADL (Table 2) due to MSDs. The percent of disability within the MSDs group was higher among those who self-reported upper back pain (80%), low back pain (74.5%), shoulder pain (66.3%), neck pain (64.3%), elbow pain (63.6%), knee pain (63.2%), hip/thigh (61.9%), and wrist/hand pain (59%).

**Table 2:** Distribution of self-reported disability of basic-activities of daily living (B-ADL) due to MSDs among the sweepers with MSDs in the past 12 months (*n* = 308)

**Variables**	**Total sample**	**MSDs related B-ADL disability**	
	**n ( %)**	**Disabled**	**Not-disabled**
All MSDs case	308 (100)	199 (64.6)	109 (35.4)
Neck pain	42 (13.6)	27 (64.3)	15 (35.7)
Shoulder pain	92 (29.9)	61 (66.3)	31 (33.7)
Upper back pain	5 (1.6)	04 (80)	01 (20)
Elbow pain	22 (7.1)	14 (63.6)	08 (36.4)
Wrist/hand pain	39 (12.7)	23 (59)	16 (41)
Low back pain	216 (70.1)	161 (74.5)	55 (25.5)
Hip/thigh pain	21 (6.8)	13 (61.9)	08 (38.1)
Knee pain	117 (38)	74 (63.2)	43 (36.8)
Ankle/foot	54 (17.5)	07 (13)	47 (87)

#### Regression analysis

Explanatory variables that were significantly associated in bivariate logistic regression (*p* < 0.25) were fitted to multivariate logistic regression analysis. After controlling potential confounding variables such as age being older than 35 years old, BMI being obese, job satisfaction being dissatisfied and cleaning distance greater than or equal to two kilometers per day were significantly associated with MSDs among street cleaners (*p* < 0.05). While variables such as, marital status, being alcoholic, and year of working experience were not significantly associated in multivariate analysis (*p* > 0.05).

The odds of developing MSD were 2.534 times higher in participants aged 35 and above (AOR: 2.534, 95% CI (1.507–4.260)) than in the 18–34 age group. Participants who were obese were 4.910 times (AOR: 4.910, 95% CI (2.216–10.877) more likely to develop MSD than those who were non-obese (Table 3). The interaction effect of age group ≥ 35, being overweight/obese, and cleaning distance of > 2 km per day was not significantly associated with self-reported MSDs.

**Table 3:** Factors associated with MSD on bivariate and multivariate logistic regression analysis of study participants among street cleaners (*n* = 422)

**Variables**	**MSD**		**Odds ratio**	
	**Yes**	**No**	**COR (95% CI)**	**AOR (95% CI)**
Age				
18 – 34 years	86	61	1 ref	1 ref
≥ 35 years	222	53	2.971 (1.90, 4.63)	2.534 (1.51, 4.26)**
Marital status				
Married	128	63	1 ref	1 ref
Single	27	18	0.738 (0.38, 1.44)	1.152 (0.547, 2.42)
Divorced	92	25	1.811 (1.06, 3.09)	1.744 (0.97, 3.13)
Widowed	61	08	3.753 (1.69, 8.32)	2.62 (1.11, 6.24)
Alcohol habits				
Yes	127	35	1.584 (1.02, 2.50)	1.07 (0.94, 2.61)
No	181	79	1 ref	1 ref
BMI kg/m2				
Normal	219	106	1 ref	1 ref
Overweight/obese	89	08	5.385 (2.52, 11.51)	4.91 (2.22, 10.87)**
Job satisfaction				
Satisfied	10	13	1 ref	1 ref
Not-satisfied	298	101	3.836 (1.63, 9.02)	2.66 (1.05, 6.75)*
Work experience				
< 10 years	249	103	1 ref	1 ref
≥ 10 years	59	11	2.219 (1.12, 4.39)	1.23 (0.57, 2.62)
Cleaning distance/day				
1-2 Km	50	42	1 ref	1 ref
> 2 km	258	72	3.010 (1.85, 4.89)	2.82 (1.64, 4.83)**

^*^significance < 0.05

^**^significance < 0.01

*CI* confidence interval, *COR* crude odds ratio, *AOR* adjusted odds ratio

## Discussion

In the modern world, MSDs are one of the major occupational health concern and exist in a wide range of occupations, particularly in low-wage physical labor, dirty work, and jobs considered underprivileged like street sweeping or waste cleaning [24]. In the current study, we sought to determine the burden of musculoskeletal disorders among street sweepers/cleaners, disability in B-ADL related to MSDs, and potential factors associated with the past 12-month MSDs. MSDs are a serious health problem in terms of the enormity of their burden, and health expenditure. With the context of expected aging, lack of findings beyond the western world [9], meager diagnostic capacity, and poor utility of health insurance, the findings of this study will contribute to the understanding of the work-related exposures and potential factors associated with one of the risk group who is often exposed to occupational hazards, the street sweepers /cleaners in a developing country.

It is noteworthy that all the participants in this study were females and non-smokers, which are potential risk factors associated with MSDs as reported in the literature. Hence, the findings of this study can therefore be used as control data for variables like female gender and non-smoking habits in a similar population. In Ethiopia, men hold the most authority and the traditional thinking dictates that cleaning/sweeping is solely the responsibility of women and cleaning, sweeping, or cooking are a shameful thing for a man, which could be a major reason for exclusive women participants. The overall annual prevalence of MSDs in any body part region among the street sweepers/cleaners was 73% (95% CI, 68.5, 77.2), and that of the previous 7 days was 59.77%. Among those with MSDs 3/5th of the street sweepers reported disability related to basic ADL during pain, and respondents were more disabled due to pain in the upper back, low back, and shoulder. The disability rate and regions involved in this study were similar to the studies elsewhere. The prevalence rate reported in this study is on the higher side of the global range for the prevalence of MSDs (26%—89%) among the working population. The high prevalence of MSDs can be attributed to LBP, and exclusive female participants in this study, about 1 out of every 2 participants in this study reported LBP which is the most prevalently reported MSD by all populations. Exposure-gender interactions, social roles of females, differential pain experience, psychology, and care-seeking might be reasons for higher reporting of pain among women.

Further, the higher estimates of MSDs are likely to be influenced by over-reporting, which may be driven by the workload, females being responsible for activity of households, gender-based low work capacity, low educational attainments, negativity associated with low wages, and working with dirt. These findings corroborate the results of previous studies. These findings corroborate with the results of studies conducted in Nigeria [25], India [26], Vietnam [27], Thailand [28], Korea [7], and Iran [29] with the observed prevalence of 78.2%, 72%, 74.4%, 88%, 69.4%, and 93% respectively. In contrast, a lower prevalence was observed in the studies conducted in Malaysia (54.5%), Egypt (60.8%), Brazil (60%), Australia (58.1%), Mekelle, Ethiopia (53.28%), and India (Chandigarh 56.8, Himachal Pradesh 58.9 [30–36]. There is a wide variance in the reported prevalence rate across the region and countries. This could be due to the variations in the definition of MSDs, the work environment, facilities, workload, and characteristics of the street sweepers studied. For instance, the Australian study [33] reported prevalence based on the data from WRMSDS claims as a part of safe of Australia to evaluate the workplace risk management practice. The Ethiopian study [34] reported point prevalence among females and literate participants were higher than our study. Further, higher educational status and occupational health safety (OHS) training reported in the other studies would have led to higher reporting of pain.

As conceptualized in our literature review [8, 9, 11, 24, 33], it is not surprising that we observed that the bodily pain increased monotonically with age, except for the upper back, hip/thigh pain, elbow pain, and neck pain, which plateaus after 46–50 years old. Given that the older working-age sample reported sensitivity to MSDs, further research is warranted to understand how age-related degenerative changes interact with the imbalance between job exposures, job demands and work capacity. The subset of low back pain contributed 51.1 percent (n 216/422) of the MSDs burden in this study, and research in the past including the GBD 2019 [9] has estimated that 37% of all global back pain reporting is attributed to occupational exposure or hazards. There is no surprise because door-to-door waste collection, loading at the collection point, and sweeping roads is a work needing repeated movements and force.

The higher illiteracy rate (41%) amongst the street sweepers observed in this study is similar to those observed in most of the countries [25, 27, 30, 31]. Further, there are some significant predictor variables identified in the logistic model, such as higher age, being overweight/obese, not-satisfied with the job, and cleaning long distances in a day. The estimated risk odds of developing MSDs was nearly 2.5 times for age-group above 34 years, not satisfied with job, cleaning > 2 km per day, and a relatively higher risk of near 5 folds if the respondents were overweight/obese. The potential risk factors found in this current study is consistent with the studies conducted elsewhere in the world. However, the current study did not observe any association of MSDs with longer work experience and low educational status unlike other studies [26, 36, 37]. This can be explained by lesser proportion of experienced workers, and equal reporting of MSDs across educational and experience category in this study. Further, sometimes the experienced sweepers think that the presence of MSDs is part of their work hazards and they would have gained a better understanding of work-related exposure to MSDs with a way to avoid them.

This study has some limitations to be mentioned to benefit future works; minor injuries during work were not recorded, physical exercise, pace of work, and current health status were not recorded which did not allow us to rule out healthy worker effects, ergonomic factors and working environment assessment, work capacity assessments were not considered and more importantly, the cross-design of this research makes it difficult to attribute (causal relationship) sweeping or cleaning work as the cause of MSDs. Further, bias in recalling MSDs, respondents of this study carried out work manually, and since this present survey was conducted at a specific point of time, seasonal variations in the burden of MSDs were not addressed. Nevertheless, despite a few limitations, the findings of this study have exposed the prevalent self-reported MSDs, and contribute significantly important data on the burden of MSDs among sweepers/cleaners in low-middle income countries which can be used as basis for preventive plans for this susceptible population. Caution should be practiced while generalizing the findings of this study beyond the limitations and to similar occupations.

## Conclusion

This study observed a relatively high prevalence of self-reported MSDs, particularly low back pain being the most involved region, and among MSDs case most of the women experienced disability related to basic activities of daily life which raises concern, especially among women who also play a key role in household responsibility. Educational program on the potential occupational hazards, improving self-health, distribution management of workload, and periodic morbidity screening program, particularly for the aged work force would reduce the problem to a larger extend.

## Data Availability

The datasets generated and/or analyzed during the current study are not publicly available due to privacy issues. However, data will be available upon formal request to the corresponding authors.

## Abbreviations

MSDs: Musculoskeletal disorders
BMI: Body mass index
GBD: Global burden of diseases
DALYs: Disability-adjusted life years
SDI: Socio-demographic index
SSA: Sub-Saharan Africa
OA: Osteoarthritis
OHS: Occupational health safety
YLD: Years lived with disability
MSQ: Minnesota satisfaction questionnaire
AOR: Adjusted odds ratio
CI: Confidence interval
LBP: Low back pain
OR: Odds ratio
SD: Standard deviation
SPSS: Statistical package for social sciences
PPE: Personal protective equipment
